# Bottlenecks Beyond Primary Care: Patient and Healthcare Worker Perspectives on Access to Specialists, Diagnostics, and System Organisation in Poland

**DOI:** 10.3390/healthcare14070894

**Published:** 2026-03-31

**Authors:** Anna Domańska, Sabina Lachowicz-Wiśniewska, Wioletta Żukiewicz-Sobczak

**Affiliations:** 1 Department of Nutrition and Food, The Faculty of Medicine and Health Science, University of Kalisz, W. Bogusławskiego 2, 62-800 Kalisz, Poland; anna_domanska@onet.pl; 2 Department of Biotechnology and Food Analysis, The Faculty of Production Engineering, Wrocław University of Economics and Business, Komandorska 118/120, 53-345 Wrocław, Poland; 3 Department of Biological Bases of Food and Feed Technologies, The Faculty of Production Engineering, University of Life Science in Lublin, Głęboka 28, 20-612 Lublin, Poland

**Keywords:** healthcare system, Poland, patient satisfaction, accessibility, quality of care, health inequalities, primary healthcare, reform, survey

## Abstract

**Background/Objectives**: Access delays in specialist consultations and diagnostics are frequently cited as key weaknesses of the Polish healthcare system. This study aimed to identify patient- and healthcare employee-reported bottlenecks beyond primary care, focusing on access, organisational and information barriers. **Methods**: A cross-sectional survey in Primed Medical Center in Lublin (south-eastern Poland) analysed fifty eligible adult respondents (58% patients; 42% healthcare employees). Measures covered access and organisational barriers (primary care, specialists/diagnostics, out-of-hours), perceived quality, equity, and satisfaction. **Results**: Overall dissatisfaction predominated (66.0% rather/definitely dissatisfied vs. 24.0% somewhat/definitely satisfied), and 70.0% indicated that reform is needed. The most frequent constraints concerned appointment scheduling convenience (88.0%), limited specialist access (86.0%), inability to obtain timely diagnostics (80.0%), unclear guidance on where to seek help (78.0%), and low administrative efficiency (74.0%). Additional concerns included out-of-hours access (60.0% reported no immediate night help) and perceived inequity (58.0% reported unequal access; 62.0% reported unequal treatment). In contrast, primary care availability was rated positively by 78% on a qualitative scale, and physician competence by 62%. Associations with sex, age, residence, and role were significant but small to moderate. **Conclusions**: Respondents differentiate clinical competence from system performance: negative assessments cluster around organisational barriers and capacity constraints in specialist and diagnostic pathways. Improving patient navigation and information, scheduling and administrative workflows, and specialist/diagnostic capacity—while strengthening primary care coordination—may reduce delays and support more equitable, higher-quality care.

## 1. Introduction

Healthcare systems are a core component of social policy, shaping population health, perceived security, and social cohesion. The World Health Organization defines health as a state of physical, mental, and social well-being rather than merely the absence of disease. Because health is not a typical market good with a clear “price”, the primary goals of healthcare systems are to protect life and well-being, and errors in organisation or provision can have irreversible consequences. Evaluating how the system functions from the users’ perspective—particularly in terms of access and quality—is therefore essential. In this study, we focus on patient-reported perceptions of healthcare system functioning, as perceived access and quality are key determinants of service use, trust, and satisfaction. Where relevant, we also include the perspectives of healthcare personnel to contrast service-user experiences with provider-side assessments [1,2,3,4,5].

The Polish healthcare system has undergone several major reforms, moving from early insurance-based solutions influenced by the Bismarck tradition and a post-war centralised Semashko-type model to the post-1989 transformation with mandatory health insurance, pluralistic provision, and a growing role of the non-public sector [6,7,8]. Subsequent changes, including the establishment of the National Health Fund (Narodowy Fundusz Zdrowia, NFZ), clarified the rules for publicly financed benefits and reshaped hospital financing and drug reimbursement [9,10,11,12,13]. As a result, Poland operates a hybrid system that combines solidarity-based public financing with market-oriented mechanisms and an expanding private segment. While these reforms have improved the formal structure of the system, they have not fully resolved long-standing problems related to access, uneven quality, and public dissatisfaction [14,15].

System performance is commonly assessed through perceived quality of care and patient satisfaction. Quality encompasses adherence to current medical knowledge and guidelines, safety of care, and the way services are delivered, including respect for patient rights, access to information, and staff competence [16,17,18,19,20,21,22,23]. The patient–professional relationship and communication (empathy, understandable explanations, trust) are critical for the care experience. At the normative level, healthcare systems are expected to balance fairness in sharing financial burdens with responsiveness to patients’ needs and expectations [24,25]. Limited resources, however, make some form of rationing unavoidable, often via waiting times, prioritisation rules, and organizational barriers [16,17,18]. Patient rights—such as the right to information, dignity, privacy, and informed consent—remain central to perceived quality and satisfaction and are an important lens for evaluating system performance [22,24,26,27,28,29,30,31].

Accessibility is another critical dimension, commonly framed through availability, acceptability, geographic and organisational accessibility, and affordability [17,18,32]. Despite universal coverage, patients in Poland report persistent barriers, including long waiting times, limited specialist availability, administrative complexity, and reliance on out-of-pocket payments for faster diagnostics and consultations [33,34]. Measures of unmet need related to waiting times place Poland among lower-performing EU countries on access indicators [15,35,36,37]. Workforce constraints further amplify access problems, as Poland has one of the lowest physician-to-population ratios in the EU, contributing to overload and delays [14,18,38,39]. These access barriers intersect with social determinants of health, reinforcing inequalities related to socioeconomic status and place of residence [19,40,41,42], consistent with the well-described social gradient in health [1]. Although multiple studies have examined selected aspects of healthcare performance in Poland, fewer analyses combine patient and healthcare staff perspectives within the same study framework while simultaneously addressing accessibility, perceived quality, organisation/information barriers, and reform needs. A county-level approach further enables capturing territorial (urban–rural) differences within typical service pathways, which are often obscured in national aggregates.

Against this background, the present study aims to analyse the functioning of the Polish healthcare system primarily from the perspective of patients, complemented by assessments provided by healthcare personnel. The findings are interpreted with reference to selected European healthcare models and indicators to contextualise the Polish experience within broader organisational and financing arrangements [16,17,36]. References to selected EU countries are used solely to provide contextual benchmarks for key access and performance indicators; the study is not a direct cross-country comparative analysis based on parallel primary data.

## 2. Materials and Methods

### 2.1. Study Design and Study Population

The study used a cross-sectional design and an anonymous questionnaire survey (see more details in Table S1). The study’s geographical scope was limited to Primed Medical Center in Lublin (south-eastern Poland) and included both urban and rural residents; a total of fifty individuals participated. Lublin was selected intentionally as a “real-life” setting, enabling direct recruitment in healthcare facilities and the local community and allowing comparison between an urban centre and surrounding rural areas within one administrative unit. The study should be interpreted as a snapshot with potential transferability to structurally similar rather than as nationally representative evidence. 

Eligible participants were adults (≥18 years) who had contact with the Polish healthcare system either as patients or as staff employed in healthcare facilities (physicians, nurses, midwives, administrative staff, and auxiliary personnel), enabling assessment from both user and provider perspectives. Sampling was non-probability and based on convenience recruitment with purposive inclusion of key subgroups (patients vs. staff; urban vs. rural residents; broad age categories). Participants were recruited through one channel (1), facility-based recruitment in selected healthcare facilities. Exclusion criteria were age < 18 years, inability to provide informed consent, and questionnaires with substantial missing data preventing classification by key variables.

The sample was described by sex, age (18–25, 26–50, 51–65, ≥66 years), place of residence (urban/rural), and role within the healthcare system (patient, medical professional, other healthcare facility employee). The 18–25 category reflected predominantly students/young adults; it was the least represented, likely due to lower healthcare utilisation and the facility-based recruitment context, which tends to include older and/or chronically ill individuals. Place of residence was operationalised as self-reported residence type (“urban” for city/town; “rural” for village/rural locality). The role was categorised as patient, medical professional (physician, nurse, midwife), and other healthcare facility employee (administrative and auxiliary personnel).

### 2.2. Research Instruments

The research instrument was a purpose-designed questionnaire developed for this study. It comprised two sections: (1) demographic and role-related information (sex, age, place of residence, and role within the healthcare system: patient, physician, nurse/midwife, other healthcare facility employee) and (2) the main questionnaire addressing: (i) accessibility of healthcare services (PHC access, specialist consultations, diagnostic tests, night care and care outside the place of residence, and difficulties in obtaining immediate medical help), (ii) perceived quality of care (physician competence, staff engagement, treatment conditions, and modern equipment), (iii) organisation and information (clarity of guidance on where to seek help, administrative efficiency, and appointment scheduling convenience), and (iv) overall assessment of the healthcare system in Poland (overall satisfaction and perceived strengths/weaknesses).

Most items were closed-ended single-choice questions using evaluative response formats (e.g., yes/no; definitely/rather yes vs. rather/definitely no); selected items were semi-open to allow brief comments. The questionnaire was designed to capture perceptions and experiences rather than clinical outcomes; therefore, emphasis was placed on clarity, face validity, and feasibility in field conditions rather than internal consistency indices for a single scale.

Questionnaire domains and items were developed to reflect key experiential dimensions of health system performance (accessibility, perceived quality, organisation/information, and overall assessment). Content validity was enhanced through iterative review by the research team with expertise in public health and healthcare organisations. Before the main survey, the questionnaire underwent pilot testing in a small convenience group of adults meeting inclusion criteria to assess comprehensibility, response burden, and clarity of key terms; minor revisions were introduced to clarify wording and standardise response options across sections.

### 2.3. Study Procedure

The survey was conducted between 1 December 2021 and 1 December 2022 at selected healthcare facilities and among residents of Lublin. Questionnaires were distributed in person by the researcher or designated staff and were completed independently by respondents. Where feasible, the research team monitored recruitment to avoid over-concentration in a single site and to include diverse participant profiles (sex, age categories, urban/rural residence, and role within the healthcare system).

Participation was voluntary. Each participant received information about the study’s aim, the anonymous and confidential nature of the survey, and that the results would be used exclusively for scientific purposes. To minimise response bias and social desirability, respondents completed the questionnaire without the presence of direct supervisors or clinical decision-makers, and completed questionnaires were returned in a manner ensuring confidentiality (e.g., sealed envelope and/or collection box, where applicable). Completion of the questionnaire was considered to constitute informed consent to participate. No personal identifiers were collected, ensuring complete anonymity.

### 2.4. Statistical Analysis

Given the heterogeneity of roles (patients vs. healthcare staff), analyses were conducted overall and stratified by role; role was also included as a grouping variable in χ^2^ tests of independence to examine differences in assessments between service users and providers. The collected data were analysed using descriptive statistics. For each questionnaire item, response frequencies and percentages (%) were calculated for individual response categories. The results are presented in tables and figures describing the study sample (sex, age, place of residence, and role within the healthcare system) and responses regarding service accessibility, quality of care, treatment conditions, system organisation, and the overall assessment of the healthcare system in Poland. Given the non-probability sampling design, the analyses are intended to characterise patterns of responses within the study sample rather than to provide nationally generalisable estimates.

Questionnaires with substantial missing data were excluded before analysis (n = 133). The remaining analyses were conducted on complete responses available for each item (complete-case per analysis).

Cramér’s V was interpreted using commonly applied thresholds (0.10 small, 0.30 medium, 0.50 large), while emphasising practical relevance alongside *p*-values. Given the exploratory nature of the analyses and the number of statistical tests performed, no formal adjustment for multiple comparisons was applied; therefore, *p*-values should be interpreted cautiously and alongside effect sizes and consistency of patterns across related items.

Analyses were performed using Statistica 13.5 (Krakow, Poland); spreadsheets were used only for data entry and cleaning. Given the county-level, non-probability sampling design, inferential findings should be interpreted as indicative associations within the study sample rather than population estimates for Poland.

## 3. Results

### 3.1. Characteristics of the Study Population

A total of fifty adults who had contact with the Polish healthcare system participated in the study. The distribution by sex was 56% women and 44% men (Table 1). The largest age group comprised respondents aged 26–50 years (34%), followed by those aged 51–65 years (24%) and >66 years (22%); the smallest age group was 18–25 years (20%). With regard to place of residence, 54% of respondents reported living in rural areas (village) and 46% in urban areas (city/town). In terms of role within the healthcare system, patients constituted 58% of the sample, while 42% were healthcare employees (including medical professionals and other facility staff) (Table 1).

### 3.2. Availability of Health Care Services

Respondents’ perceived access to a primary care physician was assessed using both a dichotomous item and a qualitative rating scale (Table 2 and Table 3). In the dichotomous question, 46% reported being able to see a PHC physician without major difficulties, while 54% reported difficulties (Table 2). In the qualitative rating of PHC availability, responses were predominantly positive: 50% rated access as “completely positive” and 28% as “somewhat positive”. Negative ratings accounted for 16%, and 6% selected “I have no opinion” (Table 2).

**Figure 1:**
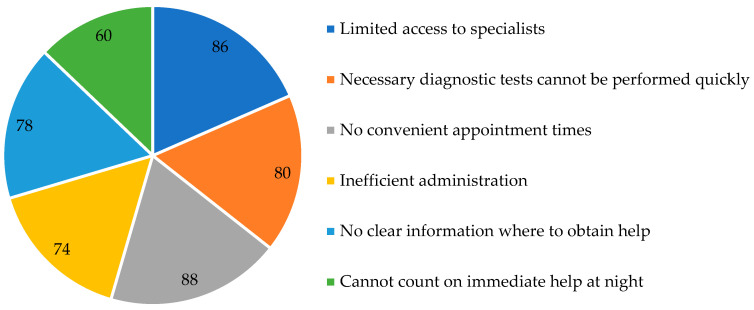
Major access and organisational problems (negative responses, %).

Perceived access to specialist care was markedly less favourable. As shown in Table 3 and Figure 1, 86% reported limited access to specialist doctors. In the qualitative assessment of the availability of specialist consultations and diagnostic tests, negative responses predominated, as 38% selected “somewhat negative” and 46% “completely negative,” whereas positive ratings were infrequent (2% “completely positive” and 6% “somewhat positive”) (Table 2). A similarly unfavourable pattern was observed for diagnostic testing. Only 20% of respondents reported that necessary diagnostic tests can be performed quickly and without major difficulties, while 80% responded “no” (95% CI for “no”: 67.0-88.8) (Table 2 and Table 3).

Night-time access was assessed in a dichotomous question. Forty percent of respondents declared that they could count on immediate medical help at night, whereas 60% reported that they could not (95% CI for “no”: 46.2-72.4) (Table 2 and Table 3). Emergency medical help was rated on an ordinal scale. Positive evaluations totalled 44% (18% “completely positive” and 26% “rather positive”), while negative evaluations totalled 54% (26% “rather negative” and 28% “completely negative”); 2% reported no opinion (Table 2).

Spatial accessibility was assessed through perceived convenience of facility locations and the possibility of obtaining care outside one’s place of residence (Table 2 and Table 3). Most respondents (66%; 95% CI: 52.2-77.6) considered doctors’ practices and diagnostic units to be located in convenient places, while 34% disagreed (Table 2 and Table 3). In addition, 70% of respondents (95% CI: 56.2-80.9) reported that it is easy to obtain medical care outside their place of residence, whereas 30% reported difficulties (Table 2 and Table 3).

Equity was assessed with two related questions. When asked whether access to publicly funded services is the same for all patients, 26% responded “yes,” 58% responded “no” (95% CI for “no”: 44.2-70.6), and 16% selected “I have no opinion” (Table 2 and Table 3). In a separate item concerning equal treatment of patients, 38% agreed that patients are treated equally, whereas 62% disagreed (Table 2).

### 3.3. Quality of Services and Competence of Medical Staff

**Figure 2:**
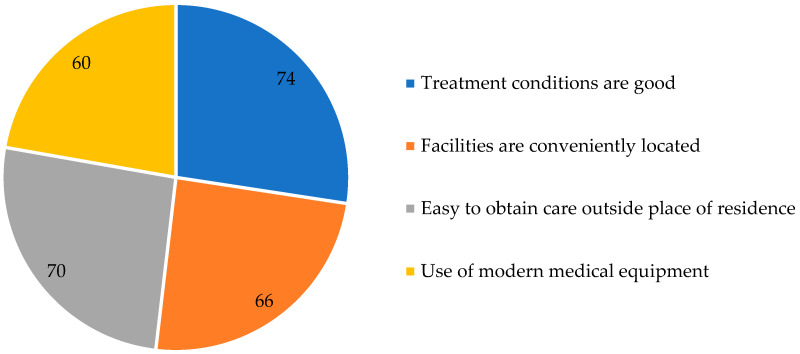
Key positive aspects (positive responses, %).

**Figure 3:**
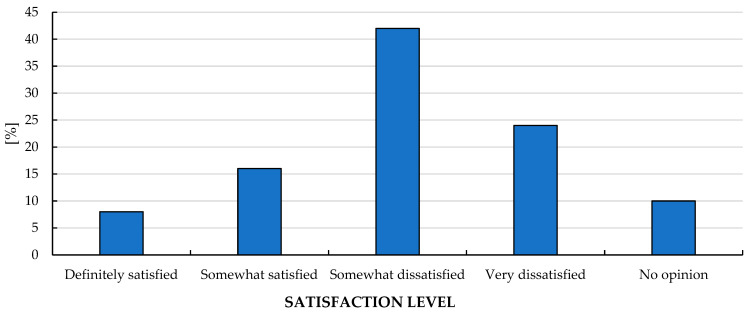
Overall satisfaction with the healthcare system in Poland.

Perceived physician competence was rated positively by 62% of respondents and negatively by 38%. In contrast, perceived engagement was evaluated more critically: 34% reported that doctors’ commitment is sufficient, while 66% disagreed. Key positive aspects of the system, as perceived by respondents, are shown in Figure 2. Perceptions of interpersonal treatment were mixed. Forty-six percent of respondents stated that patients are treated with care and kindness, whereas 54% disagreed (95% CI for “no”: 40.4–67.0). Use of modern medical equipment was reported by 60% of respondents and denied by 40%. In the qualitative assessment of equipment modernity, 6% rated it as “fully positive,” 40% as “somewhat positive,” 38% as “somewhat negative,” 12% as “fully negative,” and 4% selected “I have no opinion” (Figure 3).

A key communication-related item concerned the clarity and effectiveness of information on where to obtain medical advice or help. Only 22% responded positively, whereas 78% responded negatively (95% CI for “no”: 64.8–87.2) (Table 2 and Table 3). Responses also reflected reservations regarding equity and interpersonal aspects of care, as shown by the predominance of negative responses in items addressing equal treatment (62% “no”) and care/kindness (54% “no”) (Table 2 and Table 3).

Overall conditions of treatment were rated positively by 74% of respondents and negatively by 26% (95% CI for “yes”: 60.4–84.1) (Table 2 and Table 3). Overall quality of treatment was assessed on a qualitative scale: 10% rated it as “completely positive,” 46% as “somewhat positive,” 30% as “somewhat negative,” and 14% as “completely negative” (Table 2).

### 3.4. Organisation and Administration of the System

Administrative functioning of healthcare facilities was rated critically. Only 26% reported that administration works quickly and efficiently, whereas 74% disagreed (95% CI for “no”: 60.4–84.1) (Table 2 and Table 3). The prevalence of major access and organisational problems is summarised in Figure 1. A broader organisational rating (“efficiency of patient service and general conditions of care”) also showed predominantly negative evaluations: 6% “completely positive,” 20% “somewhat positive,” 28% “somewhat negative,” 44% “completely negative,” and 6% “no opinion” (Table 2).

Scheduling visits at convenient times was evaluated as a major organisational barrier. Only 12% reported that it is possible to arrange visits at a time convenient for the patient (without taking time off work or school), whereas 88% disagreed (95% CI for “no”: 76.2–94.4) (Table 2 and Table 3).

Perceived use of modern organisational solutions (e.g., the Internet and e-health tools) was divided: 48% responded “yes” and 52% “no” (Table 2). Administrative innovativeness was rated more negatively on the qualitative scale: 10% rated it “fully positive,” 24% “somewhat positive,” 36% “somewhat negative,” 26% “fully negative,” and 4% reported no opinion (Table 2).

### 3.5. Costs, Financing, and Co-Payments

When asked whether medical treatment in Poland is free of charge, 56% responded “yes” and 44% “no” (Table 2).

Out-of-pocket payments were rated negatively by most respondents (Table 2). Forty-one percent assessed the level of additional payments as “completely negative” and 30% as “somewhat negative,” while 27% expressed satisfaction (total positive) and 6% reported no opinion.

Respondents most frequently identified combined financial and allocation issues as the main source of problems (Table 2). The option “insufficient financial resources and poor use of funds” was selected by 48%. Additional responses included “insufficient financial resources” (26%), “poor use of funds” (18%), “the system has no problems” (4%), and “it is hard to say” (4%).

### 3.6. Overall Assessment of System Functioning and the Need for Reform

Overall satisfaction was predominantly negative (Table 2 and Table 3). In the four-category item, 8% were “definitely satisfied,” 16% “somewhat satisfied,” 42% “somewhat dissatisfied,” and 24% “very dissatisfied,” while 10% had no opinion (Table 2). Combined indicators showed that 66.0% were rather/definitely dissatisfied (95% CI: 52.2–77.6), whereas 24.0% were somewhat/definitely satisfied (95% CI: 14.3–37.4) (Table 3). The distribution of overall satisfaction categories is illustrated in Figure 1.

Most respondents indicated that the healthcare system requires reform (Table 2 and Table 3). Seventy percent responded “yes” (95% CI: 56.2–80.9), 10% responded “no,” and 20% had no opinion (Table 2).

Respondents most frequently supported introducing partial payment for all services under universal health insurance (58%; Table 2). Other options included increasing taxes/health insurance contributions (18%), abolishing the mandatory contribution with full individualisation (8%), leaving the system in its current form (8%), and “no opinion” (8%).

### 3.7. Relationships Between Socio-Demographic Characteristics and System Evaluation

Given the exploratory nature of the study, associations with sociodemographic characteristics were examined only for a limited set of key outcomes. Associations between selected sociodemographic characteristics and assessments of the healthcare system are summarised in Table 4. Overall assessment of the system’s functioning was significantly associated with gender (*p* = 0.04; Cramér’s V = 0.10). Difficulties in accessing specialist doctors were significantly associated with age (*p* = 0.009; Cramér’s V = 0.13). Diagnostic problems were significantly associated with place of residence (*p* = 0.00007; Cramér’s V = 0.16). Assessment of staff competence differed significantly between patients and healthcare workers (*p* = 0.000017; Cramér’s V = 0.17). Cross-tabulations indicated that dissatisfaction with system functioning was more frequent among older respondents, rural residents, and patients compared with healthcare workers, and that rural residents more often reported difficulties related to specialist access and diagnostics. Overall, effect sizes (Cramér’s V) ranged from 0.10 to 0.17, indicating small effect sizes (Table 4).

## 4. Discussion

The present findings indicate that the functioning of the Polish healthcare system is evaluated predominantly negatively, with overall dissatisfaction clearly prevailing. Importantly, negative assessments appear to be driven mainly by system organisation and access bottlenecks rather than uniformly poor perceptions of clinical competence. This distinction indicates actionable reform targets at the service-delivery level (patient navigation, scheduling, and referral workflows, front-office processes) and at the system level (specialist/diagnostic capacity and equity). By combining patient and staff perspectives within a county-level setting, the study highlights where perceived gaps concentrate along real-life care pathways, which may inform locally implementable improvements. This pattern is consistent with Public Opinion Research Centre (CBOS) reports, where approximately three-quarters of respondents expressed a negative assessment of the system [43,44]. Overall, respondents perceive a need for structural and organisational improvements, consistent with evidence indicating reform needs in financing, accessibility, and quality of care [43,45]. The associations observed in cross-tabulations further suggest that critical perceptions occur across multiple subgroups defined by sex, age, place of residence, and role in the system (Table 4), indicating that dissatisfaction is not confined to a single demographic profile. Beyond confirming high levels of dissatisfaction reported in national surveys, this study contributes a more granular, county-level perspective that combines the views of both patients and healthcare workers within the same analytical framework. This dual perspective highlights where perceptions converge and diverge along actual care pathways and helps identify concrete organisational “bottlenecks” that may not be fully visible in national aggregates. Such local diagnoses can guide targeted improvements at the county level while also informing debates on system-wide reforms.

A central message of this study is the distinction respondents make between clinical competence and the experience of access and service organisation. Physician competence was rated more positively than other dimensions, whereas physician engagement was assessed critically, and interpersonal experiences were mixed. This aligns with evidence that satisfaction depends not only on clinical standards but also on communication, counselling, and interpersonal treatment [2,21,22,23]. From a practical perspective, quality improvement should therefore extend beyond clinical performance indicators and include strengthening patient dialogue and counselling in routine care. Evidence from primary care nursing practice underscores the value of relationship building, empathy, maintaining patient agency, and jointly setting realistic goals to support cooperation and satisfaction [46,47]. At the same time, respondents’ ambivalence regarding care and kindness, together with negative assessments of equal treatment, corresponds to analyses indicating that patient rights—particularly dignity, privacy, and equal treatment—may not be fully respected in everyday practice [22,31].

Accessibility emerged as the most problematic domain, especially beyond primary care. In this study, PHC access was assessed more favourably on the qualitative scale than via the “without difficulties” item, suggesting that respondents differentiate between general system functioning in PHC and operational barriers related to appointment logistics. Using the accessibility framework (availability, organisational accessibility, and affordability) [17,18,32], the results show a clear gradient: PHC is rated relatively better, while specialist and diagnostic pathways form the main bottleneck. This is consistent with PHC as the system “gateway” [16,17,46,47]. It suggests that organisational accessibility (queues, scheduling capacity, referral pathways, diagnostic routing) primarily drives negative perceptions, even when spatial accessibility is rated more favourably. This broadly corresponds to national evidence indicating that PHC is relatively more accessible than other parts of the system and functions as a “gateway” to care [16,17,46,47]. Ministry of Health analyses similarly suggest that most patients obtain PHC appointments relatively easily [48,49]. By contrast, access to specialists and diagnostic tests was evaluated markedly worse. The high prevalence of reported difficulties in diagnostics and specialist access mirrors CBOS findings indicating frequent barriers to specialist services [50,51,52] and is compatible with arguments linking persistent bottlenecks to comparatively low health expenditure in Poland relative to other EU countries [52]. Additionally, the Ministry of Health points to rising expectations, population ageing, and rapid technological development—combined with insufficient oversight—as drivers of system pressure [51,53].

These access constraints should also be interpreted through the lens of prevention and early detection capacity in PHC. If preventive actions are repeatedly postponed during routine visits due to time pressure and competing needs, delayed diagnosis becomes more likely, and downstream demand for specialist consultations and high-cost diagnostics may increase [47]. This supports strengthening preventive pathways and team-based task allocation in PHC—for example, delegating low-risk repetitive tasks while preserving physician input for shared decision-making [46]. More broadly, integrating primary, secondary, tertiary, and quaternary prevention into routine practice remains challenging but is repeatedly identified as essential for system effectiveness [45].

Respondents rated spatial accessibility more favourably than temporal/organisational accessibility. Facility location and the ability to seek care outside one’s place of residence were generally assessed positively, although CBOS reports have previously highlighted location and distribution of facilities as perceived weaknesses in Poland (CBOS, 2016). This difference may reflect local county characteristics and different “distance/travel-time” perceptions among urban and rural populations. In conceptual terms, spatial accessibility is a key dimension of service availability and may vary territorially [33], with PHC acting as the principal entry point to the system [16,17,46,47]. By contrast, temporal accessibility and organisational convenience were evaluated very negatively: appointment scheduling at convenient times was widely perceived as unattainable, and night-time access was frequently rated as insufficient. This corresponds to national evidence showing dissatisfaction with scheduling and out-of-hours accessibility [50,51], and the literature emphasizes that emergency and night-time availability is an important component of subjective health security [43].

Organisational and administrative barriers were among the most consistently criticised elements of system functioning. Respondents reported major deficits in information about where to obtain help and strongly negative evaluations of administrative efficiency, indicating that patient navigation and front-office processes are key pain points. These findings align with quality management literature stressing that process standardisation and organisational improvement frameworks (e.g., accreditation standards, total quality management) can streamline administrative workflows and reduce unnecessary bureaucracy [26,27,28]. Notably, perceptions of “modernity” differed by domain: modern medical equipment was evaluated more favourably than modern organisational or administrative solutions, suggesting that technological capacity does not automatically translate into a better patient experience.

Equity emerged as another critical issue. A substantial proportion of respondents did not perceive access to publicly funded services as equal and questioned equal treatment, consistent with CBOS observations that timely access is often perceived as uneven [50,51]. This aligns with broader evidence that socio-economic, educational, and territorial factors shape both health status and service accessibility [19,40,41]. According to the WHO and Paszkowska, such inequalities can be reduced but are difficult to eliminate completely [1,42]. In this context, the observed urban–rural associations in access-related items may reflect structural differences in local availability and service organisation rather than isolated individual experiences.

Respondents also identified perceived causes of system dysfunction in both insufficient financial resources and inefficient allocation. This mirrors CBOS findings that inadequate financing is widely perceived [50,51] and aligns with evidence that expenditure levels and spending structure constrain efficiency and inequality reduction [43,44,52]. Importantly, reform preferences in this study were strongly oriented toward partial co-payments, which contrasts with CBOS findings where support for co-payment was much lower [50,51]. While such differences may reflect local experiences of access constraints, the policy implication is that co-payments, supplementary insurance, and payer competition are discussed as potential mechanisms to improve efficiency and quality—provided robust social safeguards and exemptions are implemented to prevent deepening inequities [50,51]. These approaches resemble selected solutions used in the Netherlands, where insurance-market mechanisms operate alongside universality and solidarity [16,17,46,47].

Finally, the strong perceived need for reform may be interpreted not only as a financing debate but also as a call to rebalance the system toward prevention and health promotion. Integrating prevention levels—from risk-factor reduction and early detection to limiting chronic disease consequences and quaternary prevention—requires stable financing, clear pathways, and coordination across PHC, specialist care, and public health [45]. A potentially practical local complement is community-oriented primary care (COPC), which integrates clinical and public health perspectives by diagnosing community needs, identifying inequalities, and designing locally tailored interventions [48,54,55,56].

From a policy and practice perspective, these findings point to several priority areas for action. Strengthening the supply and organisation of specialist and diagnostic services, improving appointment scheduling and out-of-hours arrangements, and standardising administrative and information processes could directly address the main bottlenecks identified by respondents. In addition, reinforcing the coordinating role of primary care and integrating preventive and counselling functions more systematically into routine visits may help reduce downstream demand for high-cost specialist care and diagnostics.

From a research perspective, further studies should extend this approach to other regions and employ probability sampling to improve generalisability. Longitudinal designs and multivariable analyses could clarify causal pathways between system characteristics, perceived accessibility, and satisfaction, while validated instruments combining patient- and provider-reported measures would allow more precise monitoring of changes over time.

In summary, respondents perceive major weaknesses in specialist and diagnostic accessibility, appointment scheduling, administrative efficiency, and information clarity, together with concerns about equity and aspects of patient rights implementation. At the same time, PHC is assessed relatively more favourably than specialist care, and treatment conditions and perceived clinical competence receive more positive ratings than organisational dimensions. In line with national data and the literature, these findings support the need for well-designed reforms aimed at improving access, equity, organisational performance, and the efficiency of resource use [43,44,57,58,59].

## 5. Limitations of the Study

This study has several limitations. First, the single district, non-probability design limits generalisability beyond structurally similar settings. Second, results are based on self-reported perceptions, which may be influenced by recall and response bias and do not directly measure objective system performance. Third, the analyses are descriptive and exploratory; therefore, observed associations should be interpreted cautiously and not as causal relationships.

## 6. Conclusions

In this, the single district cross-sectional survey conducted in Lublin, negative perceptions of the Polish healthcare system were prevalent and concentrated around access bottlenecks, organisational inefficiency, and perceived inequity. Overall dissatisfaction reached 66.0%, and 70.0% of respondents indicated that the system requires reform. The most critical deficits concerned specialist access (86.0%), timely diagnostics (80.0%), appointment scheduling convenience (88.0%), administrative efficiency (74.0%), and clarity of information on where to seek help (78.0%). In contrast, primary care was perceived as relatively better functioning than specialist and diagnostic pathways.

This study adds value beyond national opinion surveys by providing a local, the single district diagnosis of where perceived gaps concentrate in real-life care pathways—particularly in patient navigation, scheduling, and administrative processes—and by contrasting patient and healthcare staff perspectives within the same analytical framework. Although associations with sociodemographic factors were statistically significant, effect sizes were small to moderate, indicating that critical perceptions were widespread across groups.

Implications for policy and practice: Respondent-identified priorities include strengthening specialist and diagnostic capacity, improving appointment scheduling and administrative workflows, and implementing clearer patient-navigation pathways to support equitable access and reduce organisational barriers. Consolidating primary care as a coordinating platform for prevention and referrals may further mitigate downstream pressure on specialist and diagnostic services.

Implications for research: Future studies should extend sampling to multiple regions, apply validated instruments, and triangulate perception-based measures with objective indicators (e.g., waiting times, utilisation, and staffing levels) to better explain access bottlenecks and organisational deficits.

Given the single-region, non-probability design, findings should be interpreted as a region-level diagnosis with potential transferability to structurally similar mixed urban–rural counties rather than as nationally representative estimates for Poland.

## Figures and Tables

**Table 1:** Characteristics of research group.

Characteristics of Research Group	Categories	Response (%)	*p* *
Sex	Woman	56	0.0022
	Man	44	
Age	18–25	20	0.0414
	26–50	34	
	51–65	24	
	>66	22	
Place of residence	City	46	<0.001
	Village	54	
Role in the healthcare system	Patient	58	<0.001
	Healthcare worker	42	

*p* * confidence level.

**Table 2:** Survey responses on the Polish health care system (percentages) with chi-square test results (*p*).

Question	Response Category	Response Rate (%)	*p* *
Is it possible to see a primary care physician (POZ) without major difficulties?	yes	46	0.041
	no	54	
Do you think doctors are competent in their work?	yes	62	<0.001
	no	38	
Is the level of doctors’ commitment to their work sufficient?	yes	34	<0.001
	no	66	
Is modern medical equipment used in the health care system?	yes	60	<0.001
	no	40	
Is there clear and effective information on where to obtain medical advice or help?	yes	22	<0.001
	no	78	
Can you count on immediate medical help at night?	yes	40	<0.001
	no	60	
Are patients treated with care and kindness?	yes	46	0.041
	no	54	
Are the conditions of treatment in Poland good?	yes	74	<0.001
	no	26	
Is medical treatment in Poland free of charge?	yes	56	0.002
	no	44	
Are modern solutions (e.g., the Internet, e-health tools) used in the Polish health care system?	yes	48	0.308
	no	52	
Are doctors’ practices and diagnostic units located in convenient places?	yes	66	<0.001
	no	34	
Is it easy to obtain medical care also outside your place of residence?	yes	70	<0.001
	no	30	
Does the administration of health care facilities work quickly and efficiently?	yes	26	<0.001
	no	74	
Are patients treated equally?	yes	38	<0.001
	no	62	
Is it possible to arrange visits at a time convenient for the patient (without taking time off work or school)?	yes	12	<0.001
	no	88	
Can the necessary diagnostic tests be performed quickly and without major difficulties?	yes	20	<0.001
	no	80	
Does the health care system suffer from problems with limited access to specialist doctors?	yes	86	<0.001
	no	14	

Chi-square. Notes: Response Rate (%)—percentage of respondents selecting a given category; *p*—*p*-value. Percentages may not sum to 100 due to rounding. *p* * confidence level.

**Table 3:** Key indicators of healthcare system assessment in Poland: prevalence (%) with 95% confidence intervals (CI).

Variable	%	95% CI (%)
Overall, how satisfied are you with the functioning of the health care system in Poland? *(rather/definitely dissatisfied)*	66.0	52.2–77.6
Overall, how satisfied are you with the functioning of the Polish health care system? *(somewhat/definitely satisfied)*	24.0	14.3–37.4
Does the Polish health care system require reform? *(yes)*	70.0	56.2–80.9
Is access to publicly funded health care services the same for all patients? *(no)*	58.0	44.2–70.6
Does the health care system suffer from limited access to specialists? *(yes)*	86.0	73.8–93.0
Can the necessary diagnostic tests be performed quickly and without significant difficulties? *(no)*	80.0	67.0–88.8
Is it possible to arrange visits at a time convenient for the patient (without taking time off work or school)? *(no)*	88.0	76.2–94.4
Does the administration of health care facilities work quickly and efficiently? *(no)*	74.0	60.4–84.1
Is there clear and practical information on where to obtain medical advice or help? *(no)*	78.0	64.8–87.2
Can you count on immediate medical help at night? *(no)*	60.0	46.2–72.4
Are patients treated with care and kindness? *(no)*	54.0	40.4–67.0
Are the treatment conditions in Poland good? *(yes)*	74.0	60.4–84.1
Are doctors’ practices and diagnostic units conveniently located? *(yes)*	66.0	52.2–77.6
Is it easy to obtain medical care outside your place of residence as well? *(yes)*	70.0	56.2–80.9

Note: “yes/no” refers to responses to the survey questions; the “rather” and “definitely” categories were combined.

**Table 4:** Effect sizes (Cramér’s V) for selected variable pairs.

Correlations	*p* *	Cramér’s V
Gender × overall assessment of the system’s functioning	0.04	0.10
Age × difficulties in accessing specialist doctors	0.009	0.13
Place of residence × diagnostic problems	<0.001	0.16
Patient vs. healthcare worker × staff competence assessment	<0.001	0.17

Notes: *p*—*p*-value; Cramér’s V—effect size (0–1), where higher values indicate stronger association. *p* * confidence level.

## Data Availability

All data will be made available to interested readers upon request to the corresponding author; the data are not publicly available due to ethical restrictions.

## Supplementary Materials

The following supporting information can be downloaded at: https://www.mdpi.com/article/10.3390/healthcare14070894/s1, Table S1. Distribution of responses to survey items on the Polish healthcare system and results of Pearson’s chi-square tests (p).
