# Supplementary material for: Bottlenecks Beyond Primary Care: Patient and Healthcare Worker Perspectives on Access to Specialists, Diagnostics, and System Organisation in Poland
Source: Healthcare (Basel). 2026 Mar 31;14(7):894. doi: 10.3390/healthcare14070894 (PMC13073021; doi:10.3390/healthcare14070894)
Supplement: Supplementary file 1 [file healthcare-14-00894-s001.zip › healthcare-4148986-supplementary.pdf]

Article

Bottlenecks Beyond Primary Care: Patient and Healthcare Worker Perspectives on Access to Specialists, Diagnostics, and System Organisation in Poland

Anna Domańska[1], Sabina Lachowicz-Wiśniewska[1,2,*], Wioletta Żukiewicz-Sobczak[3,1,*]

[1] Department of Nutrition and Food, The Faculty of Medicine and Health Science, University of Kalisz, W. Bogusławskiego 2, 62-800 Kalisz, Poland; anna_domanska@onet.pl
[2] Department of Biotechnology and Food Analysis, The Faculty of Production Engineering, Wrocław University of Economics and Business; Komandorska 118/120, 53-345 Wrocław, Poland
[3] Department of Biological Bases of Food and Feed Technologies, The Faculty of Production Engineering, University of Life Science in Lublin, Głęboka 28, 20-612 Lublin, Poland
* Correspondence: s.lachowicz-wisniewska@uniwersytetkaliski.edu.pl (S.L.-W.); w.zukiewicz-sobczak@up.lublin.pl (W.Ż.-S.)

Table S1. Distribution of responses to survey items on the Polish healthcare system and results of Pearson's chi-square tests (p).

Question	Response Category	Response Rate (%)	*p**
How do you rate the availability of primary care (GP) services?	Completely positive	50	<0.001
	Somewhat positive	28	
	Negative (total)	16	
	I have no opinion	6	
How do you rate the modernity of medical equipment?	Fully positive	6	<0.001
	Somewhat positive	40	
	Somewhat negative	38	
	Fully negative	12	
	I have no opinion	4	
	Fully positive	10	<0.001

Academic Editor: Firstname Lastname

Received: date
Revised: date
Accepted: date
Published: date

Citation: To be added by editorial staff during production.

Copyright: © 2025 by the authors. Submitted for possible open access publication under the terms and conditions of the Creative Commons Attribution (CC BY) license (https://creativecommons.org/licenses/by/4.0/).

How do you rate the innovativeness of administrative solutions in health care facilities?	Somewhat positive	24	
	Somewhat negative	36	
	Fully negative	26	
	I have no opinion	4	
How do you rate the medical help provided in emergency situations?	Completely positive	18	<0.001
	Rather positive	26	
	Rather negative	26	
	Completely negative	28	
	I have no opinion	2	
How do you rate the overall quality of treatment?	Completely positive	10	<0.001
	Somewhat positive	46	
	Somewhat negative	30	
	Completely negative	14	
How do you rate the level of additional out-of-pocket payments?	Completely negative	41	<0.001
	Somewhat negative	30	
	Satisfied (total positive)	27	
	I have no opinion	6	
How do you rate the efficiency of patient service and the general conditions of care (organisation)?	Completely positive	6	<0.001
	Somewhat positive	20	
	Somewhat negative	28	
	Completely negative	44	
	No opinion	6	
How do you rate the availability of specialist consultations and diagnostic tests?	Completely positive	2	<0.001
	Somewhat positive	6	
	Somewhat negative	38	
	Completely negative	46	
Overall, how satisfied are you with the functioning of the health care system in Poland?	Definitely satisfied	8	<0.001
	Somewhat satisfied	16	
	Somewhat dissatisfied	42	
	Very dissatisfied	24	
	I have no opinion	10	
Does the health care system in Poland require reform?	yes	70	<0.001
	no	10	
	I have no opinion	20	
In your opinion, what are the main sources of problems with access to and the quality of health care services?	Insufficient financial resources and poor use of funds	48	<0.001
	Insufficient financial resources	26	
	Poor use of funds	18	
	The system has no problems	4	
	It's hard to say	4	
In your opinion, how should the health care system in Poland be reformed?	Introduce partial payment for all services under universal health insurance.	58	<0.001

	Increase taxes/health insurance contributions.	18	
	Abolish the mandatory contribution – full individualization.	8	
	Leave the system in its current form.	8	
	I have no opinion	8	
Is access to publicly funded health care services the same for all patients?	yes	26	<0.001
	no	58	
	I have no opinion	16	

Notes: Values are percentages of respondents selecting each response category. p—p-value. Percentages may not sum to 100 due to rounding.
